# WSN-SLAP: Secure and Lightweight Mutual Authentication Protocol for Wireless Sensor Networks

**DOI:** 10.3390/s21030936

**Published:** 2021-01-30

**Authors:** Deok Kyu Kwon, Sung Jin Yu, Joon Young Lee, Seung Hwan Son, Young Ho Park

**Affiliations:** 1 School of Electronic and Electrical Engineering, Kyungpook National University, Daegu 41566, Korea; kdk145@knu.ac.kr (D.K.K.); darkskiln@knu.ac.kr (S.J.Y.); harry250@knu.ac.kr (J.Y.L.); sonshawn@knu.ac.kr (S.H.S.); 2 School of Electronics Engineering, Kyungpook National University, Daegu 41566, Korea

**Keywords:** mutual authentication, wireless sensor networks, BAN logic, ROR model, AVISPA

## Abstract

Wireless sensor networks (WSN) are widely used to provide users with convenient services such as health-care, and smart home. To provide convenient services, sensor nodes in WSN environments collect and send the sensing data to the gateway. However, it can suffer from serious security issues because susceptible messages are exchanged through an insecure channel. Therefore, secure authentication protocols are necessary to prevent security flaws in WSN. In 2020, Moghadam et al. suggested an efficient authentication and key agreement scheme in WSN. Unfortunately, we discover that Moghadam et al.’s scheme cannot prevent insider and session-specific random number leakage attacks. We also prove that Moghadam et al.’s scheme does not ensure perfect forward secrecy. To prevent security vulnerabilities of Moghadam et al.’s scheme, we propose a secure and lightweight mutual authentication protocol for WSNs (WSN-SLAP). WSN-SLAP has the resistance from various security drawbacks, and provides perfect forward secrecy and mutual authentication. We prove the security of WSN-SLAP by using Burrows-Abadi-Needham (BAN) logic, Real-or-Random (ROR) model, and Automated Verification of Internet Security Protocols and Applications (AVISPA) simulation. In addition, we evaluate the performance of WSN-SLAP compared with existing related protocols. We demonstrate that WSN-SLAP is more secure and suitable than previous protocols for WSN environments.

## 1. Introduction

**Figure 1:**
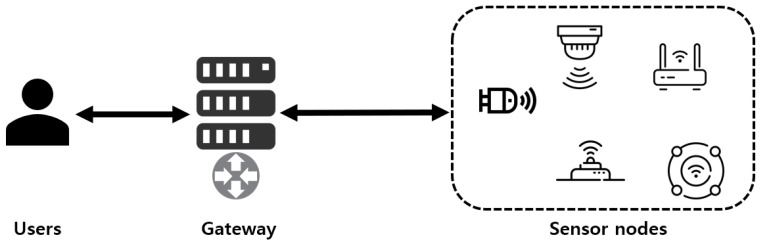
System model in Wireless sensor networks (WSNs).

As a rapid development of wireless communication technology, wireless sensor networks (WSN) can be applied to various environments such as smart grids, smart homes, agriculture, industrial internet of things (IoT), and health-care [1,2,3,4,5]. People can achieve a more bountiful life by utilizing WSN environments. Generally, WSN environments consist of sensor nodes, a gateway, and users, as shown in Figure 1. Sensor nodes detect and monitor their surrounding environment. Then, sensor nodes transmit the monitored data to the gateway. The gateway relays and analyzes the message between sensor nodes and users. The gateway also manages the private information of sensor nodes and users to provide secure services. Users can access the data collected by sensor nodes through the gateway.

An example of the application environment in WSN is health-care services. Wearable sensors attached to a patient analyze the health condition of the patient. Then, these sensors send the collected data to the physician. However, these services can be exposed to various security attacks because each entity exchanges information through a public channel. If an adversary intercepts messages in WSN, the adversary can disguise as a legal user and send an incorrect message to the sensor node. Moreover, if an adversary registers to the gateway as a legal entity, the adversary can try to obtain other legal user’s sensitive information. Therefore, we need an authentication protocol that can provide secure services and prevent various attacks in WSN environments.

In 2020, Moghadam et al. [6] suggested an authentication and key agreement scheme for WSN environments utilizing Elliptic-Curve Diffie-Hellman (ECDH) [7]. They demonstrated that their scheme is efficient and secure against various security attacks such as replay, password guessing, stolen verifier, and man-in-the-middle (MITM) attacks. However, we discover that Moghadam et al.’s scheme does not provide security against insiders, and session-specific random number leakage attacks. We also prove that Moghadam et al.’s scheme does not support perfect forward secrecy. Moreover, each entity performs Elliptic Curve Cryptography (ECC) multiplication operations to compute a session key in Moghadam et al.’s scheme. However, ECC requires heavy computational costs. Since sensor nodes have low computation capabilities and storage resources in a WSN environment, we cannot ensure real-time communications using ECC in WSN environments. Therefore, using Moghadam et al.’s scheme makes it difficult to provide efficient services. To improve security vulnerabilities and reduce the computational cost of Moghadam et al.’s scheme, we propose a secure and lightweight mutual authentication protocol (WSN-SLAP) considering security and efficiency features using hash functions and XOR operations.

### 1.1. Contributions

Our paper’s contributions are as below.

- We analyze and prove the security vulnerabilities of Moghadam et al.’s scheme. Then, we propose WSN-SLAP to resolve security vulnerabilities of Moghadam et al.’s scheme.
- We demonstrate the mutual authentication of WSN-SLAP using Burrows–Abadi–Needham (BAN) logic [8].
- We proof the session key security of WSN-SLAP by using the Real-or-Random (ROR) model [9]
- We use Automated Verification of Internet Security Protocols and Applications (AVISPA) [10,11] to prove security features of WSN-SLAP against replay and MITM attacks.
- We analyze the communication cost, the computational cost, and security properties of WSN-SLAP compared with related schemes.

### 1.2. Adversary Model

WSN-SLAP uses a well-known adversary model called the Dolev–Yao (DY) model [12]. Through the DY model, the adversary can eavesdrop, delete, intercept, and insert exchanged messages through a public channel. Moreover, the adversary can get exposed session-specific ephemeral parameters, which is based on the Canetti–Krawczyk (CK) adversary model [13]. The adversary can perform various security attacks with the DY model and the CK model. The detailed assumptions of the adversary model are defined in the following manner.

- If an adversary registers as a legal user to the gateway, the adversary can authenticate with other entities.
- An adversary can obtain a user’s lost/stolen smart card. The adversary can perform the power analysis attack [14] to get stored parameters of the smart card.
- An adversary can attempt various attacks such as replay, sensor node capture, stolen verifier, and off-line password guessing attacks.

### 1.3. Organization

In Section 2, we describe related works for WSN environments. Then, we revisit Moghadam et al.’s scheme in Section 3 and prove the security flaws of Moghadam et al.’s scheme in Section 4. Section 5 illustrates WSN-SLAP. In Section 6, we perform informal and formal security analyses of WSN-SLAP by using BAN logic, the ROR model, and AVISPA simulation tool. In Section 7, we analyze WSN-SLAP’s performance compared with the existing related protocols. In Section 8, we conclude and summarize our paper.

## 2. Related Works

In the past few decades, numerous password-based authentication schemes have been proposed to provide security and efficiency in WSN environments [15,16,17,18,19]. In 1981, Lamport [20] suggested an authentication mechanism based on a password. Lamport used one-way hash functions to encode the password and stored the hashed password inside the system. In 2006, Wong et al. [21] suggested a password-based authentication scheme in WSN environments. Unfortunately, Tseng et al. [22] proved that Wong et al.’s scheme is insecure against forgery and replay attacks. Tseng et al. demonstrated a dynamic user authentication scheme to improve security vulnerabilities of Wong et al. [21]’s scheme. However, these schemes [20,21,22] can suffer from on/off-line password guessing attacks because they only used the password as a factor to login and authenticate with other entities.

In the last few decades, two-factor-based authentication schemes [23,24,25] have been presented using hash functions and XOR operations to improve single factor’s security weaknesses. In 2009, Das et al. [23] proposed a two-factor authentication scheme based on a smart card in WSNs. They demonstrated that their scheme can prevent various attacks such as replay, stolen verifier, and off-line password guessing attacks. However, Khan et al. [24] analyzed that Das et al. [23]’s scheme is vulnerable to privileged insider attack. He et al. [25] found that Das et al. [23]’s scheme is vulnerable to insider and impersonation attacks. To improve the security vulnerabilities of Das et al.’s scheme, He et al. [25] suggested an enhanced two-factor user authentication scheme for WSNs. However, these schemes [23,24,25] can suffer from various attacks such as thoe using stolen smart cards and mobile devices.

To resolve the security flaws associated with two-factor-based authentication schemes and improve the security level in WSN environments, researchers have proposed many ECC-based authentication schemes [26,27,28,29,30,31]. In 2011, Yeh et al. [26] proposed an authentication protocol for WSN environments using ECC. Yeh et al.’s scheme used a smart card and ECC to prevent various security issues such as insider, and masquerade attacks. Choi et al. [27] suggested an ECC-based user authentication scheme for WSN. However, Wu et al. [28] pointed out that Choi et al.’s protocol does not provide security against forgery attack. Nam et al. [29] suggested a secure authentication protocol for WSN based on ECC. Nam et al.’s scheme provides a secure protocol based on an Elliptic Curve Computation Diffie-Hellman (ECCDH) problem. In 2016, Jiang et al. [30] proposed an ECC-based authentication scheme. Jiang et al.’s scheme provides secure communications and untraceability in WSN environments. In 2017, Wu et al. [31] suggested a user authentication scheme using ECC. Wu et al.’s scheme can preserve user privacy in WSN environments. However, sensor nodes in WSN have low computing power and resources. Therefore, it is difficult to provide efficiency in WSN environments using these schemes [26,27,28,29,30,31] because ECC requires large computational resources.

In 2020, Moghadm et al. [6] suggested an authentication and key agreement scheme using ECDH. They asserted that their scheme provides resistance against various attacks such as replay, MITM, off-line password guessing, and stolen verifier attacks. However, we discover that Moghadam et al.’s scheme is vulnerable to insider, session-specific random number leakage attacks and perfect forward secrecy. Moreover, Moghadam et al.’s scheme suffers from heavy computational cost because it involves an ECC-based computation. Therefore, we propose WSN-SLAP, which has resistance to various security problems.

## 3. Review of Moghadam et al.’s Scheme

Moghadam et al. proposed an authentication scheme based on ECDH in WSN [6]. Moghadam et al.’s scheme is composed of sensor node registration, user registration, and login and authentication phases. Table 1 indicates the notations of Moghadam et al.’s scheme and WSN-SLAP.

### 3.1. Sensor Node Registration Phase

**Figure 2:**
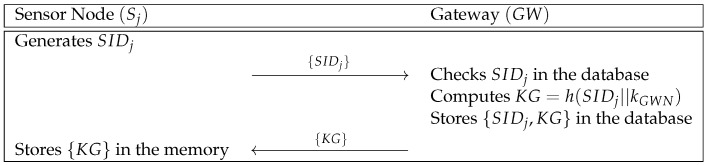
Sensor node registration phase of Moghadam et al.’s scheme.

In this phase, a sensor node $S_{j}$ sends its identity to the gateway GW. Then, GW computes a shared secret parameter between GW and $S_{j}$. In Figure 2, we show the sensor node registration phase and the details are as follows.

**Step 1:**  $S_{j}$ generates its identity $SID_{j}$, and sends it to GW over a secure channel.
**Step 2:**  GW receives $SID_{j}$ and checks the validity of $SID_{j}$. After that, GW computes $KG=h(SID_{j}||k_{GWN})$, and stores $\left\{SID_{j},KG\right\}$ in its secure database, where $k_{GWN}$ is the master key of GW. Finally, GW sends {KG} to $S_{j}$.
**Step 3:**  $S_{j}$ receives and stores {KG} in its database.

### 3.2. User Registration Phase

**Figure 3:**
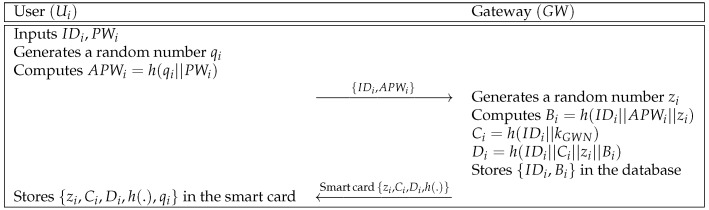
User registration phase of Moghadam et al.’s scheme.

A user $U_{i}$ registers to the gateway GW by sending an identity and a masked password value. Then, GW issues a smart card to $U_{i}$. In Figure 3, we describe the user registration phase and the details are shown as below.

**Step 1:**  $U_{i}$ inputs the identity $ID_{i}$ and the password $PW_{i}$, and then generates a random number $q_{i}$. After that, $U_{i}$ computes $APW_{i}=h(q_{i}||PW_{i})$ and sends the registration request message $\left\{ID_{i},APW_{i}\right\}$ to the gateway GW over a secure channel.
**Step 2:**  GW receives $\left\{ID_{i},APW_{i}\right\}$ from $U_{i}$, and then generates a random number $z_{i}$. After that, GW computes $B_{i}=h(ID_{i}||APW_{i}\left|\right|z_{i}),$$C_{i}=h(ID_{i}\left|\right|k_{GWN})$, and $D_{i}=h(ID_{i}\left|\right|C_{i}\left|\right|z_{i}\left|\right|B_{i})$. Finally, GW stores $\{z_{i},C_{i},D_{i},$h(.)} in a smart card and issues it to $U_{i}$ over a secure channel.
**Step 3:**  $U_{i}$ receives the smart card, and stores $q_{i}$ in the smart card. Finally, parameters $\left\{z_{i},C_{i},D_{i},h\left(.\right),q_{i}\right\}$ are stored in the smart card.

### 3.3. Login and Authentication Phase

**Figure 4:**
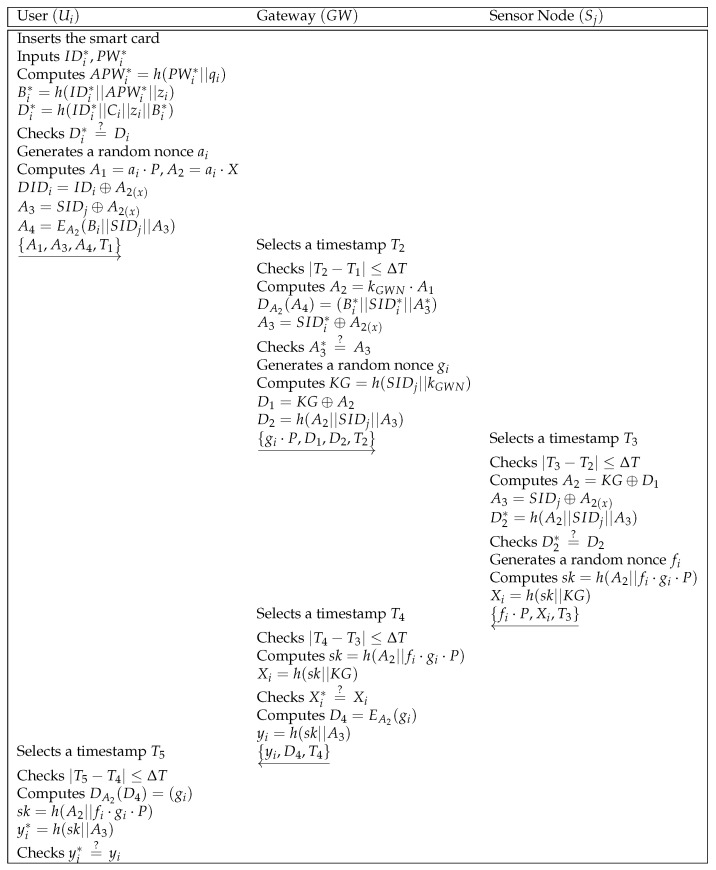
Login and authentication phase of Moghadam et al.’s scheme.

After the registration phase, the user $U_{i}$ authenticates the gateway GW. In Figure 4, we describe the login and authentication phase and the detailed steps of the phase are shown as below.

**Step 1:**  After inserting the smart card, $U_{i}$ inputs the identity $ID_{i}^{*}$ and the password $PW_{i}^{*}$. The smart card computes $APW_{i}^{*}=h(PW_{i}^{*}\left|\right|q_{i}),B_{i}^{*}=h(ID_{i}^{*}||APW_{i}^{*}\left|\right|z_{i}),D_{i}^{*}$$=h(ID_{i}^{*}||C_{i}||z_{i}||B_{i}^{*})$ and verifies $D_{i}^{*}\;\overset{?}{=}\;D_{i}$. If the verification process is successful, the smart card generates a random nonce $a_{i}$ and timestamp $T_{1}$. With the public key of the gateway *X*, the smart card computes $A_{1}=a_{i}\cdot P,A_{2}=a_{i}\cdot X,DID_{i}=ID_{i}⊕A_{2(x)},A_{3}=SID_{j}⊕A_{2(x)}$, and $A_{4}=E_{A_{2}}(B_{i}||SID_{j}\left|\right|A_{3})$. At last, the smart card sends $\left\{A_{1},A_{3},A_{4},T_{1}\right\}$ to GW through a public channel.
**Step 2:**  GW receives $\left\{A_{1},A_{3},A_{4},T_{1}\right\}$ from $U_{i}$, and selects a timestamp $T_{2}$ and checks the validity of $T_{1}$. If the timestamp is vaild, GW computes $A_{2}=k_{GWN}\cdot A_{1},D_{A_{2}}\left(A_{4}\right)=(B_{i}^{*}||SID_{i}^{*}\left|\right|A_{3}^{*}),A_{3}=SID_{i}^{*}⊕A_{2(x)}$ and verifies $A_{3}^{*}\;\overset{?}{=}\;A_{3}$. If the equality holds, GW generates a random nonce $g_{i}$ and computes $KG=h(SID_{j}||k_{GWN}),$$D_{1}=KG⊕A_{2},D_{2}=h(A_{2}||SID_{j}\left|\right|A_{3})$. At last, GW sends $\{g_{i}\cdot P,D_{1},D_{2},$$T_{2}\}$ to the sensor node $S_{j}$ over a public channel.
**Step 3:**  After reception of the message $\left\{g_{i}\cdot P,D_{1},D_{2},T_{2}\right\}$ from GW, $S_{j}$ selects a timestamp $T_{3}$ and checks the validity of $T_{2}$. Then, $S_{j}$ computes $A_{2}=KG⊕D_{1},A_{3}=SID_{j}⊕A_{2(x)},D_{2}^{*}=h(A_{2}||SID_{j}\left|\right|A_{3})$ and verifies $D_{2}^{*}\;\overset{?}{=}\;D_{2}$. If the verification is legitimate, $S_{j}$ generates a random nonce $f_{i}$, and computes $sk=h(A_{2}\left|\right|f_{i}\cdot g_{i}\cdot P),X_{i}=h\left(sk||KG\right)$. At last, $S_{j}$ sends $\left\{f_{i}\cdot P,X_{i},T_{3}\right\}$ to GW.
**Step 4:**  After receiving $\left\{f_{i}\cdot P,X_{i},T_{3}\right\}$ from $S_{j}$, GW selects a timestamp $T_{4}$ and checks the validity of $T_{3}$. Then, GW computes $sk=h(A_{2}\left|\right|f_{i}\cdot g_{i}\cdot P),X_{i}=h\left(sk||KG\right)$ and verifies $X_{i}^{*}\;\overset{?}{=}\;X_{i}$. If it is equal, GW computes $D_{4}=E_{A_{2}}\left(g_{i}\right),y_{i}=h(sk||A_{3})$ and sends $\left\{y_{i},D_{4},T_{4}\right\}$ to $U_{i}$.
**Step 5:**  $U_{i}$ receives the message $\left\{y_{i},D_{4},T_{4}\right\}$, and selects a timestamp $T_{5}$ and checks the validity of $T_{4}$. At last, $U_{i}$ computes $D_{A_{2}}\left(D_{4}\right)=\left(g_{i}\right),sk=h(A_{2}\left|\right|f_{i}\cdot g_{i}\cdot P),y_{i}^{*}=h(sk||A_{3})$ and verifies $y_{i}^{*}\;\overset{?}{=}\;y_{i}$. If it is equal, the key agreement is successful.

## 4. Cryptanalysis of Moghadam et al.’s Scheme

In this section, we demonstrate the security vulnerabilities of Moghadam et al.’s scheme [6] such as insider, and session-specific random number leakage attacks. Moghadam et al.’s scheme also does not achieve perfect forward secrecy.

### 4.1. Insider Attack

If an adversary A ordinary registers as a legal user $U_{i}$, A can authenticate with the gateway GW and the sensor node $S_{j}$ by exchanging messages. With this information, A can compute another legal user $U_{i}^{l}$’s session key. The details are shown as below.

**Step 1:**  A inserts the smart card, and inputs the identity $ID_{i}$ and the password $PW_{i}$ of A. Then, the smart card checks the validity of A, and sends a login request message $\left\{A_{1},A_{3},A_{4},T_{1}\right\}$ to GW. After authenticating A, GW sends $\left\{g_{i}\cdot P,D_{1},D_{2},T_{2}\right\}$ to $S_{j}$. Upon reception of the message $\left\{g_{i}\cdot P,D_{1},D_{2},T_{2}\right\}$, $S_{j}$ computes a session key sk. Then, $S_{j}$ sends the authentication response message $\left\{f_{i}\cdot P,X_{i},T_{3}\right\}$ to GW. GW computes the session key and sends $\left\{y_{i},D_{4},T_{4}\right\}$ to A. A computes the session key and obtains communication messages during the login and authentication phase.
**Step 2:**  After obtaining the message $\left\{g_{i}\cdot P,D_{1},D_{2},T_{2}\right\}$, A computes $KG=D_{1}⊕A_{2}$, where $A_{2}$ is the secret key of A using ECC and KG is a shared secret key between GW and $S_{j}$.
**Step 3:**  A intercepts a message $\left\{g_{i}^{l}\cdot P,D_{1}^{l},D_{2}^{l},T_{2}^{l}\right\}$ from the message of another legal user $U_{i}^{l}$. Since A knows KG, it can compute $A_{2}^{l}=D_{1}^{l}⊕KG$, where $A_{2}^{l}$ is the secret key of $U_{i}^{l}$.
**Step 4:**  A obtains the message $\left\{y_{i}^{l},D_{4}^{l},T_{4}^{l}\right\}$ and decrypts $D_{4}^{l}$ using the secret key $A_{2}^{l}$ of $U_{i}^{l}$. Then, A can obtain the random secret nonce $g_{i}^{l}$ of sensor node. A can compute $f_{i}^{l}\cdot g_{i}^{l}\cdot P$ by utilizing the message $\left\{f_{i}^{l}\cdot P,X_{i}^{l},T_{3}^{l}\right\}$. Finally, A compute the session key $sk^{l}=h(A_{2}^{l}\left|\right|f_{i}^{l}\cdot g_{i}^{l}\cdot P)$.

Therefore, Moghadam et al.’s scheme cannot prevent insider attacks.

### 4.2. Perfect Forward Secrecy

Moghadam et al. demonstrated that their scheme can ensure the security feature of perfect forward secrecy. However, if the adversary A gets the master key $k_{GWN}$ of the gateway GW, the adversary can compute the legal user $U_{i}$’s session key sk. The details are shown in following steps.

**Step 1:**  If A obtains the master key $k_{GWN}$, A can compute the secret key $A_{2}=k_{GWN}\cdot A_{1}$ of $U_{i}$ by utilizing the login request message $\left\{A_{1},A_{3},A_{4},T_{1}\right\}$.
**Step 2:**  When A intercepts the message $\left\{y_{i},D_{4},T_{4}\right\}$, A can decrypt $E_{A_{2}}\left(g_{i}\right)$ because $A_{2}$ is the symmetric key between the $U_{i}$ and the gateway GW.
**Step 3:**  After A obtains the message $\left\{f_{i}\cdot P,X_{i},T_{3}\right\}$, A can get $\left(A_{2},g_{i}\right)$ and $\left(f_{i}\cdot P\right)$. At last, A computes $U_{i}$’s session key $sk=h(A_{2}||f_{i}\cdot g_{i}\cdot P)$.

Consequently, Moghadam et al.’s scheme does not ensure perfect forward secrecy.

### 4.3. Session-Specific Random Number Leakage Attack

Suppose that a random nonce $a_{i}$ is disclosed to an adversary A. Using the public key *X* of the gateway GW, A can calculate $A_{2}=a_{i}\cdot X$. Then, A can compute the session key sk. The details are described as below.

**Step 1:**  After getting the parameter $A_{2}$, A captures the message $\left\{y_{i},D_{4},T_{4}\right\}$. Then, A decrypts $D_{4}=E_{A_{2}}\left(g_{i}\right)$ by using the symmetric key $A_{2}$ and obtains $g_{i}$.
**Step 2:**  A eavesdrops the message of the sensor node $S_{j}$$\left\{f_{i}\cdot P,X_{i},T_{3}\right\}$. Finally, A computes the session key $sk=h(A_{2}||f_{i}\cdot g_{i}\cdot P)$ using $f_{i}\cdot P$ in the message of $S_{j}$.

Therefore, Moghadam et al.’s scheme cannot prevent session-specific random number leakage attacks.

## 5. Proposed Scheme

We propose a secure and lightweight mutual authentication protocol for WSN environments to resolve security weaknesses of Moghadam et al.’s scheme [6]. To consider the resource-limited sensor nodes, WSN-SLAP uses hash functions and XOR operations that generate low computational overheads. WSN-SLAP is composed of sensor node registration, user registration, login and authentication, password update, and sensor node addition phases.

### 5.1. Sensor Node Registration Phase

**Figure 5:**
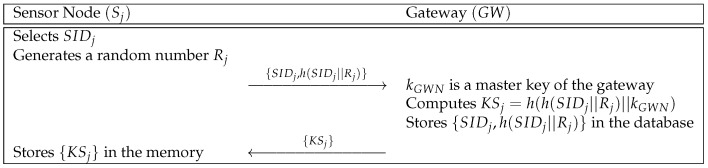
Sensor node registration phase of a secure and lightweight mutual authentication protocol (WSN-SLAP).

If a sensor node $S_{j}$ sends a registration request message, the gateway GW computes a secret parameter for the sensor node. Then, $S_{j}$ stores the parameter. We show the sensor node registration phase in Figure 5 and the details are presented as below.

**Step 1:**  $S_{j}$ selects its identity $SID_{j}$ and generates a random number $R_{j}$. Then, $S_{j}$ computes $h(SID_{j}||R_{j})$ and sends $\{SID_{j},h(SID_{j}\left|\right|R_{j})\}$ to GW over a secure channel.
**Step 2:**  GW receives $\{SID_{j},h(SID_{j}\left|\right|R_{j})\}$ and computes $KS_{j}=h(h(SID_{j}\left|\right|R_{j})||k_{GWN})$, where $k_{GWN}$ is the master key of GW. GW stores $\{SID_{j},h(SID_{j}\left|\right|$$R_{j})\}$ in the secure database and sends $\left\{KS_{j}\right\}$ to $S_{j}$.
**Step 3:**  At last, $S_{j}$ stores $\left\{KS_{j}\right\}$ in its memory.

### 5.2. User Registration Phase

**Figure 6:**
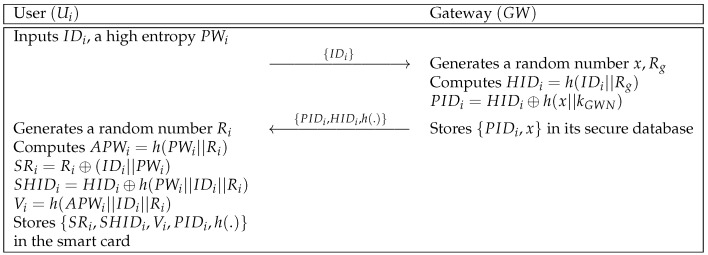
User registration phase of WSN-SLAP.

A user $U_{i}$ sends a registration request message to the gateway GW. Then, GW computes secret parameters and issues a smart card to the user. In Figure 6, we describe the user registration phase and the detailed steps are shown as below.

**Step 1:**  $U_{i}$ inputs an identity $ID_{i}$ and a high entropy password $PW_{i}$. After that, $U_{i}$ transmits $\left\{ID_{i}\right\}$ to GW via a secure channel.
**Step 2:**  GW generates random numbers *x* and $R_{g}$, and computes $HID_{i}=h(ID_{i}\left|\right|R_{g}),\;$$PID_{i}$$=HID_{i}⊕h(x||k_{GWN})$. GW stores $\left\{PID_{i},x\right\}$ in its secure database and sends the message $\left\{PID_{i},HID_{i},h\left(.\right)\right\}$ to $U_{i}$.
**Step 3:**  $U_{i}$ generates a random number $R_{i}$. With $R_{i}$, $U_{i}$ computes $APW_{i}=h(PW_{i}\left|\right|R_{i}),\;$$SR_{i}=R_{i}⊕(ID_{i}||PW_{i}),\;SHID_{i}=HID_{i}⊕h(PW_{i}||ID_{i}\left|\right|R_{i})$, and $V_{i}=h($$APW_{i}$$||ID_{i}\left|\right|R_{i})$. Finally, $U_{i}$ stores $\left\{SR_{i},SHID_{i},V_{i},PID_{i},h\left(.\right)\right\}$ in the smart card.

### 5.3. Login and Authentication Phase

**Figure 7:**
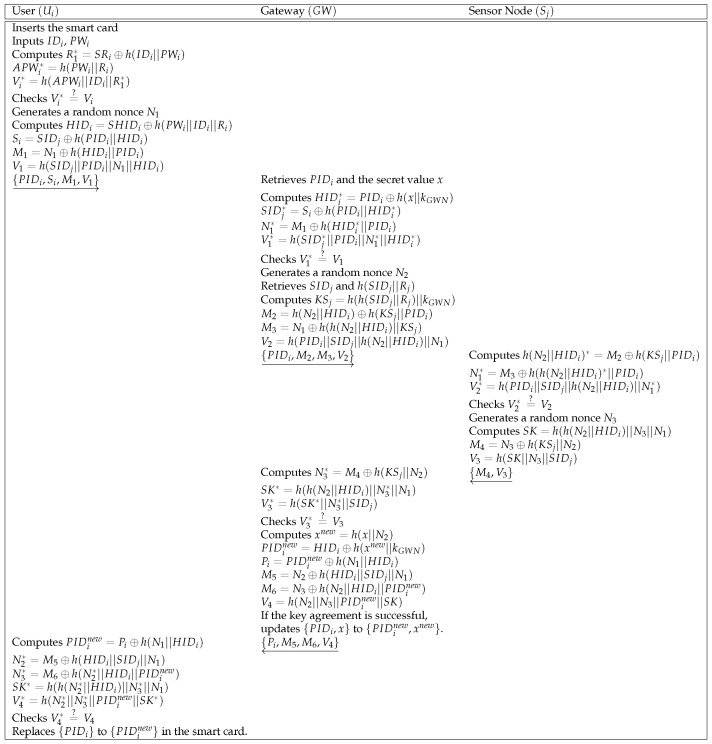
Login and authentication phase of WSN-SLAP.

To access information of the sensor $S_{j}$, the user $U_{i}$ sends a login request message to the gateway GW. In Figure 7, we describe the login and authentication phase and the details are presented below.

**Step 1:**  After inserting the smart card, $U_{i}$ inputs the identity $ID_{i}$ and the password $PW_{i}$. The smart card computes $R_{1}^{*}=SR_{i}⊕h(ID_{i}||PW_{i}),APW_{i}^{*}=h(PW_{i}\left|\right|R_{i})$ and $V_{i}^{*}=h(APW_{i}^{*}||ID_{i}\left|\right|R_{1}^{*})$. Then, the smart card checks the validity of $V_{i}^{*}$ compared with $V_{i}$ stored in the smart card. If the validity is confirmed, the smart card generates a random nonce $N_{1}$, and computes $HID_{i}=SHID_{i}⊕h(PW_{i}||ID_{i}\left|\right|R_{i}),S_{i}=SID_{j}⊕h(PID_{i}||HID_{i}),M_{1}=N_{1}⊕h(HID_{i}||PID_{i}),$ and $V_{1}=h(SID_{j}||PID_{i}\left|\right|N_{1}||HID_{i})$. At last, $U_{i}$ sends $\left\{PID_{i},S_{i},M_{1},V_{1}\right\}$ to GW over a public channel.
**Step 2:**  When GW receives $\left\{PID_{i},S_{i},M_{1},V_{1}\right\}$ from $U_{i}$, GW retrieves $PID_{i}$ and the shared secret value *x* from GW’s database. Then, GW computes $HID_{i}^{*}=PID_{i}⊕h(x||k_{GWN}),SID_{j}^{*}=S_{i}⊕h(PID_{i}\left|\right|$$HID_{i}^{*}),N_{1}^{*}=M_{1}⊕h(HID_{i}^{*}||PID_{i})$ and $V_{1}^{*}=h(SID_{j}^{*}||PID_{i}\left|\right|N_{1}^{*}||HID_{i}^{*})$, and checks the validity of $V_{1}^{*}$ compared with $V_{1}$. If the validity is confirmed, GW retrieves $SID_{j}$ and $h(SID_{j}||R_{j})$ from GW’s database. GW computes $KS_{j}=h(h(SID_{j}\left|\right|R_{j})||k_{GWN}),M_{2}=h(N_{2}||HID_{i})⊕h(KS_{j}||PID_{i}),$$M_{3}=N_{1}⊕h(h(N_{2}||HID_{i})||KS_{j}),$ and $V_{2}=h(PID_{i}||SID_{j}||h(N_{2}||HID_{i})||N_{1})$. At last, GW sends $\left\{PID_{i},M_{2},M_{3},V_{2}\right\}$ to $S_{j}$ over a public channel.
**Step 3:**  If $S_{j}$ receives $\left\{PID_{i},M_{2},M_{3},V_{2}\right\}$, $S_{j}$ computes $h(N_{2}||HID_{i}{)}^{*}=M_{2}⊕h(KS_{j}\left|\right|$$PID_{i}),N_{1}^{*}=M_{3}⊕h(h(N_{2}||HID_{i}{)}^{*}\left|\right|PID_{i}$),$V_{2}^{*}=h(PID_{i}||SID_{j}||h(N_{2}||HID_{i})||$$N_{1}^{*})$ and checks the validity of $V_{2}^{*}$ compared with the parameter $V_{2}$. If the validity is confirmed, $S_{j}$ computes $SK=h(h(N_{2}||HID_{i})||N_{3}\left|\right|N_{1}),M_{4}=N_{3}⊕h(KS_{j}\left|\right|N_{2}),V_{3}=h(SK||N_{3}$$||SID_{j})$, where SK is a session key. Finally, $S_{j}$ sends $\left\{M_{4},V_{3}\right\}$ to GW.
**Step 4:**  After receiving the message $\left\{M_{4},V_{3}\right\}$ from $S_{j}$, GW computes $N_{3}^{*}=M_{4}⊕h(KS_{j}\left|\right|N_{2}),SK^{*}=h(h(N_{2}||HID_{i})||N_{3}^{*}\left|\right|N_{1}),V_{3}^{*}=h(SK^{*}\left|\right|N_{3}^{*}\left|\right|$$SID_{j})$ and verifies the equality of $V_{3}^{*}$ and $V_{3}$. If the verification is successful, GW generates a random nonce $N_{2}$ and computes $x^{new}=h(x||N_{2}),PID_{i}^{new}=HID_{i}⊕h(x^{new}\left|\right|k_{GWN}),P_{i}=PID_{i}^{new}⊕h(N_{1}||HID_{i}),M_{5}=N_{2}⊕h(HID_{i}||SID_{j}\left|\right|$$N_{1})$$,M_{6}=N_{3}⊕h(N_{2}\left|\right|HID_{i}$$||PID_{i}^{new})$ and $V_{4}=h(N_{2}\left|\right|N_{3}\left|\right|$$PID_{i}^{new}\left||SK\right)$. At last, GW sends $\left\{P_{i},M_{5},M_{6},V_{4}\right\}$ to $U_{i}$ and updates $\left\{PID_{i},x\right\}$ to $\left\{PID_{i}^{new},x^{new}\right\}$ if the key agreement is successful.
**Step 5:**  When $U_{i}$ receives the message $\left\{P_{i},M_{5},M_{6},V_{4}\right\}$ from GW, $U_{i}$ computes $PID_{i}^{new}$$=P_{i}⊕h(N_{1}||HID_{i}),N_{2}^{*}=M_{5}⊕h(HID_{i}||SID_{j}\left|\right|N_{1}),N_{3}^{*}=M_{6}⊕h(N_{2}^{*}\left|\right|HID_{i}$||$PID_{i}^{new})$$,SK^{*}=h(h(N_{2}^{*}||HID_{i})||N_{3}^{*}\left|\right|N_{1}),V_{4}^{*}=h(N_{2}^{*}\left|\right|N_{3}^{*}\left|\right|$$PID_{i}^{new}||SK^{*})$ and checks the validity of $V_{4}^{*}$ compared with $V_{4}$. If the validity is confirmed, $U_{i}$ replaces $\left\{PID_{i}\right\}$ to $\left\{PID_{i}^{new}\right\}$ in the smart card.

### 5.4. Password Update Phase

In WSN-SLAP, users can easily change their own password. The details are shown as below.

**Step 1:**  After inserting the smart card, The user $U_{i}$ inputs the identity $ID_{i}$ and the password $PW_{i}$. The smart card computes $R_{i}^{*}=SR_{i}⊕h(ID_{i}||PW_{i}),APW_{i}^{*}=h(PW_{i}\left|\right|R_{i}),V_{i}^{*}=h(APW_{i}||ID_{i}\left|\right|R_{i}^{*})$ and verifies the equality of $V_{i}^{*}$ and $V_{i}$. If the verification is successful, the smart card requests a new password to $U_{i}$.
**Step 2:**  $U_{i}$ inputs a new password $PW_{i}^{new}$. The smart card selects a random number $R_{i}^{new}$ and computes $APW_{i}^{new}=h(PW_{i}^{new}\left|\right|R_{i}^{new}),SR_{i}^{new}=R_{i}^{new}⊕(ID_{i}||PW_{i}^{new})$$,SHID_{i}^{new}=HID_{i}⊕h(PW_{i}^{new}||ID_{i}\left|\right|R_{i}^{new}),V_{i}^{new}=h(APW_{i}^{new}||ID_{i}\left|\right|R_{i}^{new})$. Finally, the smart card stores $\left\{SR_{i}^{new},SHID_{i}^{new},V_{i}^{new},PID_{i},h\left(.\right)\right\}$.

### 5.5. Sensor Node Addition Phase

To add a new sensor node $S_{j}^{new}$ to WSN-SLAP, $S_{j}^{new}$ registers to the gateway GW. The detailed steps are described as follows.

**Step 1:**  $S_{j}^{new}$ selects its identity $SID_{j}^{new}$. Then, $S_{j}^{new}$ generates a random number $R_{j}^{new}$. With $SID_{j}^{new}$ and $R_{j}^{new}$, $S_{j}^{new}$ computes $h(SID_{j}^{new}||R_{j}^{new})$ and sends $\{SID_{j}^{new},h($$SID_{j}^{new}$$\left|\right|R_{j}^{new})\}$ to GW through a secure channel.
**Step 2:**  After receiving $\{SID_{j}^{new},h(SID_{j}^{new}\left|\right|R_{j}^{new})\}$ from $S_{j}^{new}$, GW computes $KS_{j}^{new}=h(h(SID_{j}^{new}\left|\right|R_{j}^{new})||k_{GWN})$ and stores $\{SID_{j}^{new},h(SID_{j}^{new}\left|\right|R_{j}^{new})\}$ in the database of GW. Finally, GW sends $\left\{KS_{j}^{new}\right\}$ to $S_{j}^{new}$.
**Step 3:**  $S_{j}^{new}$ receives the message $\left\{KS_{j}^{new}\right\}$ from GW and stores $\left\{KS_{j}^{new}\right\}$ in the memory of $S_{j}^{new}$.

## 6. Security Analysis

WSN-SLAP not only considers lightweight features using hash functions and XOR operations, but also ensures a higher security level compared with related schemes. To evaluate the security of WSN-SLAP, we perform informal security analysis and formal security analysis such as BAN logic, ROR model, and AVISPA simulation tool. We show that WSN-SLAP prevents a variety of attacks using informal analysis. We demonstrate the mutual authentication of WSN-SLAP using BAN logic and also prove the session key security of WSN-SLAP by using the ROR model. We use the AVISPA simulation tool to prove security features of WSN-SLAP against replay and MITM attacks.

### 6.1. Informal Security Analysis

WSN-SLAP provides security against various attacks such as insider, stolen smart card, replay, sensor node capture, off-line password guessing, privileged insider, stolen verifier, and MITM attacks. Furthermore, WSN-SLAP ensures perfect forward secrecy and mutual authentication.

#### 6.1.1. Insider Attack

If an adversary A registers to the gateway GW as a legal user, A can authenticate to GW and the sensor node $S_{j}$. A captures messages $\left\{PID_{i},M_{2},M_{3},V_{2}\right\},\left\{M_{4},V_{3}\right\}$ and $\left\{P_{i},M_{5},M_{6},V_{4}\right\}$. Then, A computes $h(h(N_{2}||HID_{i})||KS_{j})=M_{3}⊕N_{1}$ and $h(KS_{j}||PID_{i})$$=M_{2}⊕h(N_{2}||HID_{i})$. To compromise other legal user’s sessions, A must need $KS_{j}$ to compute the session key. Since hash functions mask the random nonce $N_{2}$ and the user’s secret parameter $HID_{i}$ such as $h(h(N_{2}||HID_{i})||KS_{j})$, A cannot compute the shared secret parameter $KS_{j}$ between GW and $S_{j}$. Therefore, WSN-SLAP is secure against the insider attacks.

#### 6.1.2. Stolen Smart Card Attack

Suppose that an adversary A captures the legal user $U_{i}$’s smart card. Then, A uses the power analysis attack to extract stored parameters in the smart card. With $U_{i}$’s smart card parameters, A tries to authenticate with the gateway GW and the sensor node $S_{j}$. However, A cannot compute the login request message $\left\{PID_{i},S_{i},M_{1},V_{1}\right\}$ because $HID_{i}$ is masked by $SHID_{i}=HID_{i}⊕h(PW_{i}||ID_{i}\left|\right|R_{i})$. To calculate $HID_{i}$, A needs to guess $ID_{i}$ and $PW_{i}$ at the same time. Since these tasks are computationally infeasible task, it is hard to obtain both $ID_{i}$ and $PW_{i}$. For these reasons, WSN-SLAP is secure against stolen smart card attacks.

#### 6.1.3. Replay Attack

If an adversary A intercepts messages $\left\{PID_{i},M_{2},M_{3},V_{2}\right\}$ and $\left\{ID_{i},S_{i},M_{1},V_{1}\right\}$ from a legal user $U_{i}$, A tries to authenticate with the gateway GW by sending intercepted messages at other sessions. In WSN-SLAP, GW and the sensor node check the freshness of random nonces $N_{1},N_{2}$ and $N_{3}$. Thus, WSN-SLAP can provide security against replay attacks.

#### 6.1.4. Sensor Node Capture Attack

We assume that an adversary A captures a specific sensor node $S_{j}$ and obtains parameters $\{SID_{j},$$KS_{j}\}$ from the $S_{j}$’s memory by using the power analysis attack. Then, A can authenticate with gateway GW and user $U_{i}$. However, A cannot threat other sensor nodes. Since the shared secret parameter $KS_{j}=h(h(SID_{j}\left|\right|R_{j})||k_{GWN})$, A can only authenticate with the specific sensor node $S_{j}$. A cannot calculate any information about other sensor nodes. Therefore, WSN-SLAP is secure against sensor node capture attacks.

#### 6.1.5. Off-Line Password Guessing Attack

According to Section 1.2, an adversary A can guess a legal user $U_{i}$’s password $PW_{i}$. A can also extract stored parameters $\left\{SR_{i},SHID_{i},V_{i},PID_{i},h\left(.\right)\right\}$ from $U_{i}$’s legitimate smart card. Then, A tries to impersonate as $U_{i}$. However, A cannot compute $R_{i}=SR_{i}⊕h(ID_{i}||PW_{i})$ to obtain $HID_{i}=SHID_{i}⊕h(PW_{i}||ID_{i}\left|\right|R_{i})$ without knowing the identity $ID_{i}$. Therefore, A cannot compute the legal message $\left\{PID_{i},M_{2},M_{3},V_{2}\right\}$. Accordingly, WSN-SLAP has resistance to off-line password-guessing attacks.

#### 6.1.6. Privileged Insider Attack

If a privileged insider adversary A intercepts a legal user $U_{i}$’s registration message $\left\{ID_{i}\right\}$, A tries to compute $U_{i}$’s session key by using messages in Section 5.3. However, A cannot compute the session key of $U_{i}$. To compute $SK=h(h(N_{2}||HID_{i})||$$N_{3}\left|\right|N_{1})$, A has to calculate $HID_{i}$ which is the shared secret parameter between $U_{i}$ and the gateway GW. However, A cannot compute $HID_{i}=SHID_{i}⊕h(PW_{i}||ID_{i}\left|\right|R_{i})$ from the login request message $\left\{PID_{i},S_{i},M_{1},V_{1}\right\}$ without $U_{i}$’s password and the random number $R_{i}$. Consequently, WSN-SLAP ensures security against privileged insider attacks.

#### 6.1.7. Stolen Verifier Attack

Assuming that an adversary A steals the gateway GW’s verification table including $\{SID_{j},h(SID_{j}\left|\right|R_{j})\}$ and $\left(PID_{i},x\right)$. However, A cannot compute the session key of the legal user $U_{i}$ with these parameters. To compute the session key $SK=h(h(N_{2}||HID_{i})||N_{3}\left|\right|$$N_{1})$, A must compute $HID_{i}$ by using $PID_{i}=HID_{i}⊕h(x||k_{GWN})$. Since the parameter $k_{GWN}$ is GW’s master key, A cannot compute $HID_{i}$. Therefore, WSN-SLAP has resistance to stolen verifier attacks.

#### 6.1.8. MITM Attack

During the login and authentication phase, an adversary A intercepts and tries to modify the login request message $\left\{PID_{i},S_{i},M_{1},V_{1}\right\}$. However, the gateway GW can easily detect the modified message by using the verification table. In addition, it is impossible to modify all messages because they include random parameters. Therefore, WSN-SLAP can prevent MITM attacks.

#### 6.1.9. Session-Specific Random Number Leakage Attack

Assume that an adversary A obtains all random parameters $N_{1},N_{2}$, and $N_{3}$. Then, A tries to compute the session key SK. However, it is impossible to calculate the session key without knowing $HID_{i}$. $HID_{i}$ is masked with the secret key *x* and the master key $k_{GWN}$ during the session. Accordingly, WSN-SLAP is secure against session-specific random number leakage attacks.

#### 6.1.10. Perfect Forward Secrecy

We suppose that an adversary A obtains GW’s master key $k_{GWN}$. Then, A tries to compute the session key $SK=h(h(N_{2}||HID_{i})||N_{3}\left|\right|N_{1})$ of the user $U_{i}$. However, the master key $k_{GWN}$ is utilized, i.e., $h(x||k_{GWN})$ and $h(h(SID_{j}\left|\right|R_{j})||k_{GWN})$. Therefore, A needs the shared secret parameter *x* or $h(SID_{j}||R_{j})$ to analyze the secret parameter. For this reason, WSN-SLAP provides perfect forward secrecy.

#### 6.1.11. Mutual Authentication

To authenticate with each other, each participant of WSN-SLAP performs verification processes. The gateway GW checks the validity of $V_{1}\;\overset{?}{=}\;V_{1}^{*}$ and $V_{3}\;\overset{?}{=}\;V_{3}^{*}$, the sensor node $S_{j}$ verifies $V_{2}\;\overset{?}{=}\;V_{2}^{*}$, and the $U_{i}$ checks $V_{4}\;\overset{?}{=}\;V_{4}^{*}$. If the whole verification process is successful, we can conclude that each participant is authenticated with each other. Therefore, WSN-SLAP guarantees mutual authentication.

### 6.2. BAN Logic

In this section, we prove mutual authentication of WSN-SLAP using BAN logic analysis [8]. BAN logic has been widely used to analyze the mutual authentication of various authentication schemes [32,33]. In WSN-SLAP, the participants authenticate with each other to establish a session key SK among *U*, GW, and SN. Table 2 presents the basic notations of the BAN logic used in this proof.

#### 6.2.1. Rules

The logical rules of the BAN logic are described as below.

**1.**  Message meaning rule (MMR):
  $$\frac{P_{1}\;|\equiv P_{1}\overset{Key}{↔}P_{2},\;P_{1}⊲{\left(S_{1}\right)}_{Key}}{P_{1}\left|\equiv P_{2}\right|∼S_{1}}$$
**2.**  Nonce verification rule (NVR):
  $$\frac{P_{1}\left|\equiv \#\left(S_{1}\right),\;P_{1}\right|\equiv P_{2}\;|∼S_{1}}{P_{1}\left|\equiv P_{2}\right|\equiv S_{1}}$$
**3.**  Jurisdiction rule (JR):
  $$\frac{P_{1}|\equiv P_{2}|⟹S_{1},\;P_{1}\left|\equiv P_{2}\right|\equiv S_{1}}{P_{1}\;|\equiv S_{1}}$$
**4.**  Belief rule (BR):
  $$\frac{P_{1}\;|\equiv \left(S_{1},S_{2}\right)}{P_{1}\;|\equiv S_{1}}$$
**5.**  Freshness rule (FR):
  $$\frac{P_{1}\;|\equiv \#\left(S_{1}\right)}{P_{1}\;|\equiv \#\left(S_{1},S_{2}\right)}$$

#### 6.2.2. Goals

In WSN-SLAP, the basic goals of the BAN logic are that each principal establishes a session key and achieves mutual authentication. The goals for proving mutual authentication of WSN-SLAP are defined as follows:

**Goal 1:**  $U|\equiv U\overset{SK}{↔}GW$
**Goal 2:**  $U|\equiv GW|\equiv U\overset{SK}{↔}GW$
**Goal 3:**  $GW|\equiv U\overset{SK}{↔}GW$
**Goal 4:**  $GW|\equiv U\equiv U\overset{SK}{↔}GW$
**Goal 5:**  $SN|\equiv SN\overset{SK}{↔}GW$
**Goal 6:**  $SN|\equiv GW|\equiv SN\overset{SK}{↔}GW$
**Goal 7:**  $GW|\equiv SN\overset{SK}{↔}GW$
**Goal 8:**  $GW|\equiv SN|\equiv SN\overset{SK}{↔}GW$

#### 6.2.3. Idealized Forms

In WSN-SLAP, the authentication request and response messages $\left\{PID_{i},S_{i},M_{1},V_{1}\right\}$, $\left\{PID_{i},M_{2},M_{3},V_{2}\right\}$, $\left\{M_{4},V_{3}\right\}$, and $\left\{P_{i},M_{5},M_{6},V_{4}\right\}$ are transmitted through a public channel. We will transmit these messages into the idealized form and omit other messages because they cannot efficiently provide the logical properties of BAN logic. WSN-SLAP’s idealized form messages are shown as below:

$Msg_{1}$ : $U\to GW:{\left\{N_{1},SID_{j}\right\}}_{HID_{i}}$
$Msg_{2}$ : $GW\to SN:\{h(N_{2}||HID_{i}{),N_{1}\}}_{KS_{j}}$
$Msg_{3}$ : $SN\to GW:{\left\{N_{3}\right\}}_{KS_{j}}$
$Msg_{4}$ : $GW\to U:{\left\{N_{2},N_{3}\right\}}_{HID_{1}}$

#### 6.2.4. Assumptions

After the registration phase, each principal believes that it has secret keys which are shared among each other. The principal also trusts that random numbers and pseudo identity are fresh. Moreover, the principal believes that a legal principal can control the entitled components and values. The assumptions of the BAN logic in WSN-SLAP are as below:

$A_{1}$: $GW|\equiv \#(N_{1})$
$A_{2}$: $GW|\equiv \#(N_{3})$
$A_{3}$: $SN|\equiv \#(h(N_{2}||HID_{i}))$
$A_{4}$: $U|\equiv \#(N_{2})$
$A_{5}$: $U|\equiv GW\Rightarrow (U\overset{SK}{↔}GW)$
$A_{6}$: $GW|\equiv U\Rightarrow (U\overset{SK}{↔}GW)$
$A_{7}$: $SN|\equiv GW\Rightarrow (SN\overset{SK}{↔}GW)$
$A_{8}$: $GW|\equiv SN\Rightarrow (SN\overset{SK}{↔}GW)$
$A_{9}$: $U|\equiv U\overset{HID_{i}}{↔}GW$
$A_{10}$: $GW|\equiv U\overset{HID_{i}}{↔}GW$
$A_{11}$: $SN|\equiv SN\overset{KS_{j}}{↔}GW$
$A_{12}$: $GW|\equiv SNKS_{j}GW$

#### 6.2.5. BAN Logic Proof

We conduct the BAN logic analysis of WSN-SLAP as follows:

**Step 1:**  $S_{1}$ can be obtained from $Msg_{1}$.
  $S_{1}$: $GW⊲{\left\{N_{1},SID_{j}\right\}}_{HID_{i}}$
**Step 2:**  $S_{2}$ can be induced by applying the MMR using $S_{1}$ and $A_{10}$.
  $S_{2}$: $GW|\equiv U|∼(N_{1},SID_{j})$
**Step 3:**  $S_{3}$ can be induced by applying the FR using $S_{2}$ and $A_{1}$.
  $S_{3}$: $GW|\equiv \#(N_{1},SID_{j})$
**Step 4:**  $S_{4}$ can be induced by applying the NVR using $S_{2}$ and $S_{3}$.
  $S_{4}$: $GW|\equiv U|\equiv (N_{1},SID_{j})$
**Step 5:**  $S_{5}$ is can be induced by $S_{4}$ and the BR.
  $S_{5}$: $GW|\equiv U|\equiv (N_{1})$
**Step 6:**  $S_{6}$ is obtained from $Msg_{2}$.
  $S_{6}$: $SN⊲\{h(N_{2}||HID_{i}{),N_{1}\}}_{KS_{j}}$
**Step 7:**  $S_{7}$ is can be induced by applying the MMR using $S_{6}$ and $A_{13}$.
  $S_{7}:SN|\equiv GW|∼(h(N_{2}||HID_{i}),N_{1})$
**Step 8:**  $S_{8}$ is can be induced by applying the FR using $S_{7}$ and $A_{3}$.
  $S_{8}:SN|\equiv \#(h(N_{2}||HID_{i}),N_{1})$
**Step 9:**  $S_{9}$ is can be induced by applying the NVR using $S_{7}$ and $S_{8}$.
  $S_{9}:SN|\equiv GW|\equiv (h(N_{2}||HID_{i}),N_{1})$
**Step 10:**  $S_{10}$ is obtained from $Msg_{3}$.
  $S_{10}:GW⊲{\left\{N_{3}\right\}}_{KS_{j}}$
**Step 11:**  $S_{11}$ can be induced by applying the MMR using $A_{5}$ and $S_{8}$.
  $S_{11}$: $GW|\equiv SN|∼(N_{3})$
**Step 12:**  $S_{12}$ can be induced by applying the NVR using $S_{9}$ and $S_{10}$.
  $S_{12}:GW\left|\equiv SN\right|\equiv \left(N_{3}\right)$
**Step 13:**  $S_{13}$ and $S_{14}$ can be induced by $S_{9}$, and $S_{12}$. SN and GW can compute the session key $SK=h(h(N_{2}||HID_{i})||N_{3}\left|\right|N_{1})$.
  $S_{13}:GW\left|\equiv SN\right|\equiv \left(SN\overset{SK}{↔}GW\right)$ (Goal 8)
  $S_{14}:SN\left|\equiv GW\right|\equiv \left(SN\overset{SK}{↔}GW\right)$ (Goal 6)
**Step 14:**  $S_{15}$ and $S_{16}$ can be induced by applying the JR using $S_{13}$ and $A_{8}$, and $S_{14}$ and $A_{7}$, respectively.
  $S_{15}:GW|\equiv \left(SN\overset{SK}{↔}GW\right)$ (Goal 7)
  $S_{16}:SN|\equiv \left(SN\overset{SK}{↔}GW\right)$ (Goal 5)
**Step 15:**  $S_{17}$ is obtained from $Msg_{4}$.
  $S_{17}$: $U⊲{\left\{N_{2},N_{3}\right\}}_{HID_{i}}$
**Step 16:**  $S_{18}$ can be induced by $A_{9}$, $S_{17},$ and the MMR.
  $S_{18}$: $U|\equiv GW|∼(N_{2},N_{3})$
**Step 17:**  $S_{19}$ can be induced by applying the FR using $S_{18}$ and $A_{4}$.
  $S_{19}$: $U|\equiv \#(N_{2},N_{3})$
**Step 18:**  $S_{20}$ can be induced by $S_{16}$, $S_{17},$ and the NVR.
  $S_{20}$: $U|\equiv GW|\equiv (N_{2},N_{3})$
**Step 19:**  $S_{21}$ and $S_{22}$ can be induced by $S_{5}$, $S_{18}$. *U* and GW can compute the session key $SK=h(h(N_{2}||HID_{i})||N_{3}\left|\right|N_{1})$
  $S_{21}:U\left|\equiv GW\right|\equiv \left(U\overset{SK}{↔}GW\right)$ (Goal 2)
  $S_{22}:GW\left|\equiv U\right|\equiv \left(U\overset{SK}{↔}GW\right)$ (Goal 4)
**Step 20:**  $S_{23}$ and $S_{24}$ can be induced by applying the JR using $S_{21}$ and $A_{5}$, $S_{22}$, and $A_{6}$, respectively.
  $S_{23}:U|\equiv \left(U\overset{SK}{↔}GW\right)$ (Goal 1)
  $S_{24}:GW|\equiv \left(U\overset{SK}{↔}GW\right)$ (Goal 3)

### 6.3. ROR Model

This section proves the security of the session key of WSN-SLAP by using the well-known Real-Or-Random (ROR) model [9]. In WSN-SLAP, there are three participants. $P_{U}^{t_{1}}$ is a user, $P_{GW}^{t_{2}}$ is a gateway, and $P_{GW}^{t_{2}}$ is a sensor node. In the ROR model, the network is under an adversary A who can eavesdrop, capture, insert, and delete messages. With these abilities, A performs various attacks using Execute,CorruptSC,Reveal,Send, and Test queries.

- Execute: This query is a passive attack that A can eavesdrop the legal entity’s message.
- CorruptSC: This query means A obtains stored parameters from the user’s smart card.
- Reveal: This query means A reveals the session key SK.
- Send: This query is an active attack that A sends a message to receive a response message.
- Test: An adversary A obtains a flipped unbiased coin before the game starts. If A obtains c=1, it means the session key SK is fresh. If A obtains c=0, it means the session key is not fresh. Otherwise, A obtains a NULL value. To ensure the security of the session key, it is necessary that A cannot distinguish the result value between a random number and the session key.

#### Security Proof

**Theorem** **1.** *Let A attempt to obtain the session key of WSN-SLAP in polynomial time as follows. $Adv_{A}\left(Poly\right)$ is the probability of the session key being broken by A. $q_{h}^{2}$, HASH, and $q_{send}$ mean the number of hash queries, the range space of the hash function, and the number of send queries, respectively. $s^{\prime }$ and $C^{\prime }$ are the Zipf’s parameters [34].*

$$Adv_{A}\left(Poly\right)\leq \frac{q_{h}^{2}}{\left|HASH\right|}+2\left\{C^{\prime }q_{send}^{s^{\prime }}\right\}$$

*We follow the proof according to the method of [35,36]. We perform four games $Game_{k}$, where k∈[0,3]. $Succ_{A,Game_{k}}$ is the event that A can guess a correct bit c in the $Game_{k}$, and $Pr[Succ_{A,Game_{k}}]$ is the probability of $Succ_{A,Game_{k}}$. We can perform $Game_{k}$ as follows with these parameters.*

- *$Game_{0}$: This game describes a real attack of A in WSN-SLAP under the ROR model. The random bit c needs to be selected before starting the game. Therefore, we can derive as follows.*
  (1) $$Adv_{A}\left(Poly\right)=\left|2Pr\left[Succ_{A,Game_{0}}\right]-1\right|$$
- *$Game_{1}$: In the $Game_{1}$, A obtains each entity’s messages $\left\{PID_{i},S_{i},M_{1},V_{1}\right\}$, $\left\{PID_{i},M_{2},M_{3},V_{2}\right\}$, $\left\{M_{4},V_{3}\right\}$, and $\{P_{i},M_{5},M_{6},$$V_{4}\}$ using Execute query. Then, A performs Test and Reveal queries to obtain the session key SK. Since $SK=h(h(N_{2}||HID_{i})||N_{3}\left|\right|N_{1})$, A has to get random nonces $N_{1}$, $N_{2}$, and $N_{3}$. In addition, A needs the user’s masked identity $HID_{i}$. For these reasons, A cannot calculate SK. This means $Game_{0}$ and $Game_{1}$ are indistinguishable. Therefore, we can get the following equivalent.*
  (2) $$Pr\left[Succ_{A,Game_{1}}\right]=Pr\left[Succ_{A,Game_{0}}\right]$$
- *$Game_{2}$: In this game, A performs Send query, which is an active attack. A utilizes $\left\{PID_{i},S_{i},M_{1},V_{1}\right\}$, $\left\{PID_{i},M_{2},M_{3},V_{2}\right\}$, $\left\{M_{4},V_{3}\right\}$, and $\left\{P_{i},M_{5},M_{6},V_{4}\right\}$ to get the session key SK. Parameters $V_{1}$, $V_{2}$, $V_{3}$, and $V_{4}$ are masked by HASH query. In addition, parameters $PID_{i}$, $M_{1}$, $M_{2}$, $M_{3}$, $M_{4}$, $M_{5}$, $M_{6}$, and $P_{i}$ contain random nonces $N_{1}$, $N_{2}$, and $N_{3}$. By using random nonces, we can prevent collision from other sessions. According to the birthday paradox [37], we can get the following inequation.*
  (3) $$|Pr\left[Succ_{A,Game_{2}}\right]-Pr\left[Succ_{A,Game_{1}}\right]|\leq \frac{q_{h}^{2}}{\left|HASH\right|}$$
- *$Game_{3}$: In the $Game_{3}$, A executes CorruptSC query and obtains smart card’s stored parameters $\left\{SR_{i},SHID_{i},V_{i},PID_{i}\right\}$ by using the power analysis attack, where $SR_{i}=R_{i}⊕h(ID_{i}||PW_{i}),\;SHID_{i}=HID_{i}⊕h(PW_{i}||ID_{i}\left|\right|R_{i}),\;$$V_{i}=h(APW_{i}||ID_{i}\left|\right|R_{i})$, and $PID_{i}=HID_{i}⊕h(x||k_{GWN})$. To obtain $R_{i}$ and $HID_{i}$, A needs the identity $ID_{i}$ and the password $PW_{i}$. Therefore, A cannot distinguish with $Game_{2}$ and $Game_{3}$ if guessing $PW_{i}$ is computationally infeasible task. Then, we can obtain the result by using Zipf’s law [34].*
  (4) $$|Pr\left[Succ_{A,Game_{3}}\right]-Pr\left[Succ_{A,Game_{2}}\right]|\leq C^{\prime }q_{send}^{s^{\prime }}$$
*Finally, A gets the guessed bit c because games are done.*
  (5) $$Pr\left[Succ_{A,Game_{3}}\right]=\frac{1}{2}$$
*Moreover, we can get the following result by using (1) and (2).*
  (6) $$\frac{1}{2}Adv_{A}\left(Poly\right)=|Pr\left[Succ_{A,Game_{0}}\right]-\frac{1}{2}|=|Pr\left[Succ_{A,Game_{1}}\right]-\frac{1}{2}|$$
*Using (5) and (6), we obtain the following equation.*
  (7) $$\frac{1}{2}Adv_{A}\left(Poly\right)=\left|Pr\left[Succ_{A,Game_{1}}\right]-Pr\left[Succ_{A,Game_{3}}\right]\right|$$
*We get the following result utilizing the triangular inequality.*
  $$\frac{1}{2}Adv_{A}\left(Poly\right)=|Pr\left[Succ_{A,Game_{1}}\right]-Pr\left[Succ_{A,Game_{3}}\right]|\;\leq |Pr\left[Succ_{A,Game_{1}}\right]-Pr\left[Succ_{A,Game_{2}}\right]|\;+|Pr\left[Succ_{A,Game_{2}}\right]-Pr\left[Succ_{A,Game_{3}}\right]|$$
  (8) $$\leq \frac{q_{h}^{2}}{2|HASH|}+C^{\prime }q_{send}^{s^{\prime }}$$
*By multiplying (8) by 2, we get the following result.*
  $$Adv_{A}\left(Poly\right)\leq \frac{q_{h}^{2}}{\left|HASH\right|}+2\left\{C^{\prime }q_{send}^{s^{\prime }}\right\}$$
*Therefore, we prove*

### 6.4. AVISPA Simulation

In this section, we analyze security features of WSN-SLAP by using AVISPA [10,11]. AVISPA is a formal security verification tool that detects MITM and replay attacks against the authentication protocol.

AVISPA uses the High-Level Protocols Specification Language (HLPSL). After receiving a protocol written in HLPSL, the translator converts the HLPSL-based protocol to an intermediate format (IF). Then, the translator inputs the IF to four back-ends, which are Constraint Logic-based Attack Searcher (CL-AtSe), Tree Automata based on Automatic Approximations for Analysis of Security Protocol (TA4SP), SAT-based Model-Checker (SATMC), and On the fly Model-Checker (OFMC), respectively. Consequently, the IF is converted to an output format (OF). If the summary of OF is SAFE, it means the protocol has resistance to replay and MITM attacks.

Specifically, OFMC back-end can utilize XOR operations. Therefore, we use this back-end in our paper.

#### 6.4.1. HLPSL Specifications

**Figure 8:**
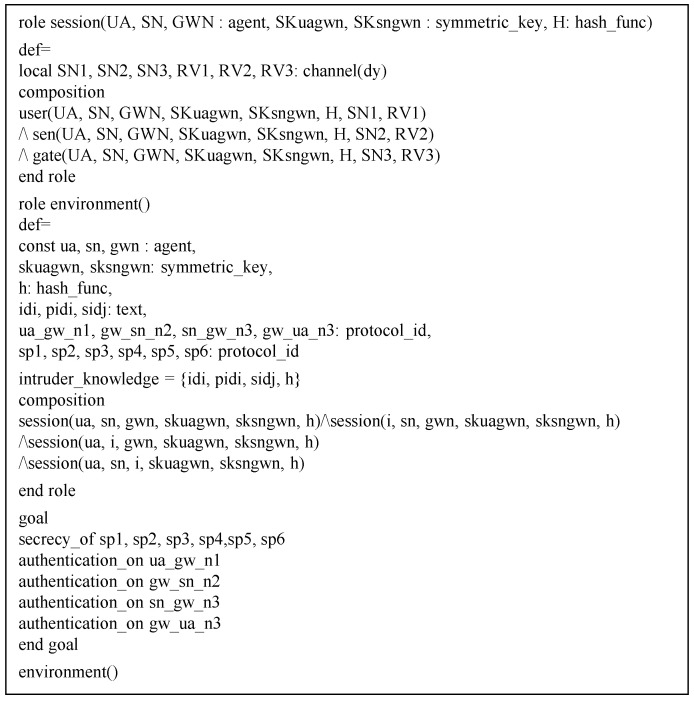
Role of session, environment and goal.

In HLPSL, WSN-SLAP consists of users UA, gateway GWN, and sensor nodes SN. These entities are written as role. There are also two compositionroles named session and environment, which contain security goals. Figure 8 indicates goals and the role of session and environment of WSN-SLAP.

**Figure 9:**
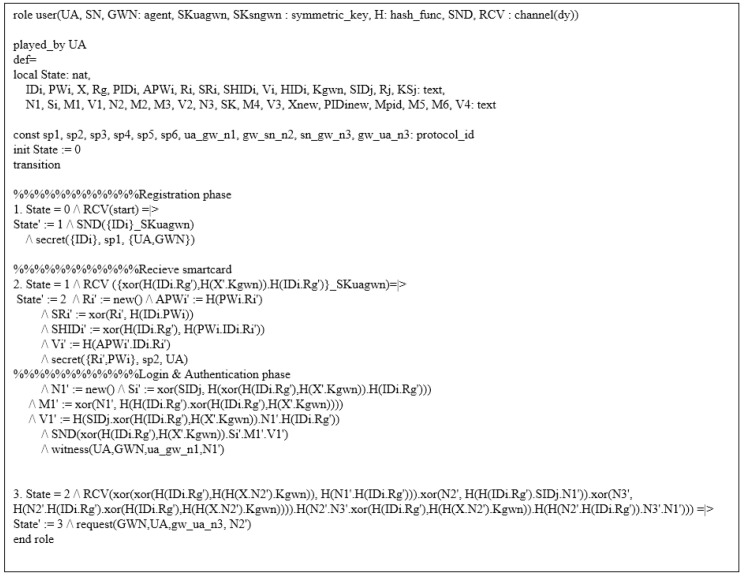
Role of user.

Figure 9 shows the whole process of the user UA. In state 1, the user UA registers to GWN. To start the session, UA receives the *start* message. Then, UA sends a registration request message $\left\{ID_{i}\right\}$ to the gateway GWN through a secure channel. In state 2, UA receives a smart card from GWN and stores $\left\{R_{i},SR_{i},SHID_{i},V_{i}\right\}$ in the smart card. In the login and authentication phase, UA sends $\left\{PID_{i},S_{i},M_{1},V_{1}\right\}$ to GWN via a public channel. The function $witness(UA,GWN,ua\_gw\_n1,N1^{\prime })$ indicates the freshness of $N_{1}$ generated by UA. In State 3, UA receives $\left\{P_{i},M_{5},M_{6},V_{4}\right\}$ from GWN. Then, UA authenticates with GWN using $N_{2}^{\prime }$ in $request(GWN,UA,gw\_ua\_n3,N_{2}^{\prime })$.

#### 6.4.2. Simulation Result

**Figure 10:**
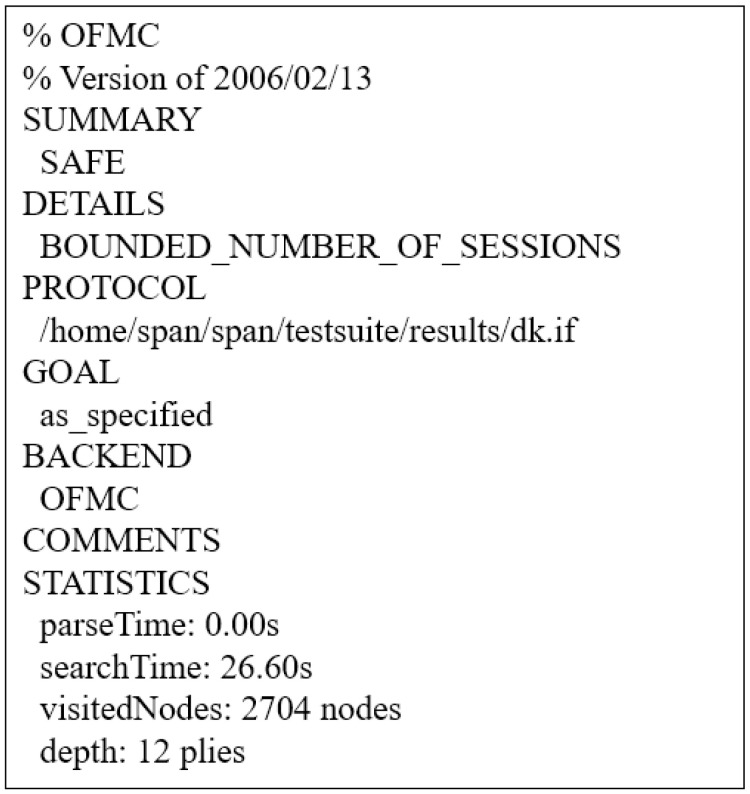
Result of the Automated Verification of Internet Security Protocols and Applications (AVISPA) simulation.

If the protocol’s result summary is SAFE in OFMC simulation, the protocol has resistance to replay and MITM attacks. The result of WSN-SLAP’s AVISPA simulation tool using OFMC back-end is shown in Figure 10. Thus, WSN-SLAP can prevent replay and MITM attacks.

## 7. Performance Analysis

In this section, we estimate computational costs, communication costs, and security properties of WSN-SLAP compared with existing related schemes [6,27,28,31].

### 7.1. Computational Costs

We analyze WSN-SLAP’s computational cost compared with the performance of the related schemes [6,27,28,31]. According to [6,38], the execution time of each operation is acquired on a computer with a four-core 3.2 GHz CPU, and 8 GB memory. We estimate that $T_{h}$, $T_{ecm}$, and $T_{sym}$ are the execution time of the hash function (≈0.00032 s), ECC point multiplication (≈0.0171 s), and symmetric encryption/decryption (≈0.0056 s), respectively. We do not consider the execution time of the XOR operation because it is negligible. Table 3 indicates the result for computational costs. Accordingly, WSN-SLAP has a more efficient computational cost than related schemes [6,27,28,31].

### 7.2. Communication Costs

We evaluate the communication cost of WSN-SLAP compared with related schemes [6,27,28,31] in this section. According to [6], we define that the user identity, sensor node identity, random number, timestamp, SHA-1 hash digest, and ECC point are 128, 16, 128, 32, 160 and 320 bits, respectively. In WSN-SLAP, the login request message $\left\{PID_{i},S_{i},M_{1},V_{1}\right\}$ requires (160+160+160+160=640 bits), and the transmitted authentication messages $\left\{PID_{i},M_{2},M_{3},V_{2}\right\}$, $\left\{M_{4},V_{3}\right\}$, and $\left\{P_{i},M_{5},M_{6},V_{4}\right\}$ require (160+160+160+160=640 bits), (160+160=320 bits), and (160+160+160+160=640 bits), respectively. Consequently, total communication costs of WSL-SLAP and related schemes [6,27,28,31] are as shown in Table 4. Therefore, WSN-SLAP provides a more efficient communication cost than related schemes do [6,27,28,31].

### 7.3. Security Properties

In Table 5, we present the security properties of WSN-SLAP with related schemes [6,27,28,31]. We show that existing protocols [6,27,28,31] suffer from various attacks, including insider, stolen smart card, and session-specific random number leakage attacks. Therefore, WSN-SLAP provides better functionality and security features compared with those of related schemes [6,27,28,31].

## 8. Conclusions

In this paper, we discovered that Moghadam et al.’s scheme has vulnerabilities against insider, and session-specific random number leakage attacks. We also proved that Moghadam et al.’s scheme does not guarantee perfect forward secrecy. To resolve the security weaknesses of Moghadam et al.’s scheme, we proposed a secure and lightweight mutual authentication protocol for WSN environments. WSN-SLAP has resistance to various attacks, including insider, stolen smart card, off-line password guessing, stolen verifier, and session-specific random number leakage attacks. We demonstrated that WSN-SLAP provides perfect forward secrecy and mutual authentication. We proved the security of WSN-SLAP using formal security analyses, which are AVISPA, BAN logic, and ROR model. Moreover, WSN-SLAP has lightweight computational and communication costs because it involves XOR operations and hash functions. Therefore, the proposed WSN-SLAP provides more secure and efficient communication services compared with existing related protocols and is suitable for WSN environments. In future work, we will implement a whole network and secure protocol to design a new scheme that is practical for use in WSN.

## Figures and Tables

**Table 1:** Notations.

Notation	Description
$U_{i}$	User
GW	Gateway
$S_{j}$	Sensor node
$ID_{i}$	Real identity of user
$PW_{i}$	Password of user
$PID_{i}$	Pseudo identity of user
$SID_{j}$	Real identity of sensor node
$k_{GWN}$	Master key of gateway
KG	Shared secret key between gateway and sensor node
*X*	Public key of gateway
*G*	Elliptic curve group
*P*	Generator of *G*
$R_{k},N_{k},z_{i},a_{i},f_{i},g_{i},q_{i}$	Random numbers
$T_{k}$	Timestamp
SK	Session key
$E_{k}/D_{k}$	Symmetric key encryption/decryption
h(.)	Hash function
||	Concatenation function
⊕	Exclusive-or function

**Table 2:** The basic notations.

Notation	Description
$P_{1},P_{2}$	Two principals
$S_{1},S_{2}$	Two statements
SK	The session key
$P_{1}|$≡$S_{1}$	$P_{1}$ believes $S_{1}$
$P_{1}|$∼$S_{1}$	$P_{1}$ once said $S_{1}$
$P_{1}$⇒$S_{1}$	$P_{1}$ controls $S_{1}$
$P_{1}$⊲$S_{1}$	$P_{1}$ receives $S_{1}$
$\#S_{1}$	$S_{1}$ is fresh
${\left\{S_{1}\right\}}_{Key}$	$S_{1}$ is encrypted with Key
$P_{1}$ $\overset{Key}{↔}$ $P_{2}$	$P_{1}$ and $P_{2}$ have shared key Key

**Table 3:** Computational costs comparison.

Schemes	User	Gateway	Sensor Node	Total	Total Cost (s)
Choi et al. [27]	$9T_{h}+3T_{ecm}$	$6T_{h}+2T_{ecm}$	$5T_{h}+1T_{ecm}$	$20T_{h}+6T_{ecm}$	0.109
Wu et al. [28]	$12T_{h}+2T_{ecm}+1T_{sym}$	$11T_{h}+2T_{sym}$	$4T_{h}+2T_{ecm}+1T_{sym}$	$27T_{h}+4T_{ecm}+4T_{sym}$	0.09944
Wu et al. [31]	$13T_{h}+2T_{ecm}$	$13T_{h}$	$4T_{h}+2T_{ecm}$	$30T_{h}+4T_{ecm}$	0.078
Moghadam et al. [6]	$5T_{h}+3T_{ecm}+2T_{sym}$	$5T_{h}+3T_{ecm}+2T_{sym}$	$3T_{h}+2T_{ecm}$	$13T_{h}+8T_{ecm}+4T_{sym}$	0.16336
Ours	$13T_{h}$	$18T_{h}$	$6T_{h}$	$37T_{h}$	0.01184

**Table 4:** Communication costs comparison.

Schemes	Communication Costs	Number of Messages
Choi et al. [27]	3200 bits	4 messages
Wu et al. [28]	3296 bits	4 messages
Wu et al. [31]	3392 bits	4 messages
Moghadam et al. [6]	2512 bits	4 messages
Ours	2240 bits	4 messages

**Table 5:** Security properties.

Security Property	Choi et al. [27]	Wu et al. [28]	Wu et al. [31]	Moghadam et al. [6]	Ours
Insider Attack	∘	∘	×	×	∘
Stolen Smart Card Attack	×	×	×	∘	∘
Replay Attack	∘	∘	∘	∘	∘
Sensor Node Capture Attack	∘	∘	∘	∘	∘
Off-line Password Guessing Attack	×	×	∘	∘	∘
Privileged Insider Attack	∘	∘	×	∘	∘
Stolen Verifier Attack	×	∘	∘	∘	∘
MITM Attack	∘	∘	×	∘	∘
Session-Specific Random Number Leakage Attack	×	×	×	×	∘
Perfect Forward Secrecy	∘	∘	∘	×	∘
Mutual Authentication	∘	∘	∘	∘	∘

∘: Secure from the attack. ×: Insecure from the attack.
